# Carotidynia Possibly due to Localized Vasculitis in a Patient with Latent *Mycobacterium tuberculosis* Infection

**DOI:** 10.1155/2013/585789

**Published:** 2013-12-02

**Authors:** Giulia Cassone, Michele Colaci, Dilia Giuggioli, Andreina Manfredi, Marco Sebastiani, Clodoveo Ferri

**Affiliations:** Chair and Rheumatology Unit, University of Modena and Reggio Emilia, Medical School, Azienda Ospedaliero-Universitaria, Policlinico di Modena, Via del Pozzo 71, 41100 Modena, Italy

## Abstract

Carotidynia is a syndrome characterized by tenderness of the carotid artery near the bifurcation due to numerous, heterogeneous causes. Here we reported the case of a 31-year-old Moroccan woman with right-sided neck pain and tenderness with irradiation to ipsilateral ear, eye, and occipital region. Clinical symptoms and imaging findings were suggestive of primary variant of carotidynia syndrome. In particular, color-Doppler ultrasonography revealed a concentric wall thickening of the distal common carotid artery, while thoracic magnetic resonance showed localized perivascular enhancement of the soft tissue in the right medial-distal common carotid artery in T1-weighted images, without intraluminal diameter variation. Moreover, careful clinicoserological and imaging investigations (cranial, cervical, and thoracic angiocomputed tomography and magnetic resonance) excluded well-known disorders potentially responsible for carotidynia syndrome. The patient was scarcely responsive to nonsteroidal anti-inflammatory drugs, but clinical symptoms resolved after three months. Of interest, the patient showed latent *Mycobacterium tuberculosis* infection (positive tuberculosis interferon-gamma release assay; QuantiFERON-TB Gold); this finding suggested a possible triggering role of mycobacterial antigens in the immune-mediated mechanism responsible for localized carotid injury.

## 1. Introduction

Carotidynia is a syndrome characterized by either unilateral or bilateral tenderness of the carotid artery near the bifurcation, first described in 1927 by Fay [1]. Generally, it has been considered as merely symptom of numerous, heterogeneous causes of neck pain (infections, migraine, trigeminal neuralgia, neoplasms, eagle syndrome, and various carotid disorders including aneurysm, dissection, occlusion, or inflammation, i.e., carotid arteritis), and less frequently as a distinct clinicopathological entity [2–4]. In 1988, the International Headache Society Classification Committee (IHSCC) published four criteria for its diagnosis: (A) at least one of the following signs overlying the carotid artery: (1) tenderness, (2) swelling, or (3) increased pulsations; (B) appropriate investigations do not reveal any structural abnormality; (C) pain over the affected side of the neck which may project to the ipsilateral side of the head; (D) a self-limiting syndrome of less than two week duration [5]. While the Headache Classification Subcommittee of IHS removed carotidynia as a distinct entity from the main classification in 2004 [6], currently, carotidynia remains a poorly understood and controversial subject with unknown ethiology [1, 7]. However, several publications have demonstrated that carotidynia can be considered as a clinical entity due to its characteristic radiological findings [7–12]. The presence of focal eccentric thickening of the carotid wall by enhancing tissue, generally without haemodynamic changes, is a typical pattern found in imaging studies in patients suffering from carotidynia [7–12]. Here we describe a patient with these peculiar clinicoradiological findings suggestive of primary variant of carotidynia syndrome.

## 2. Case Presentation

We recently observed the case of a 31-year-old Moroccan woman referring since January 2013 right-sided neck pain and tenderness with irradiation to ipsilateral ear, eye, and occipital region. Her clinical past history revealed a lymphadenitis of the neck at the age of thirteen but not drug or toxic addiction or traumatic injuries. Moreover, well-defined vascular, autoimmune, and/or metabolic (diabetes, dyslipidemia, and/or obesity) disorders were invariably ruled out, as well as other risk factors of vascular alterations such as smoke.

The patient's physical examination was unremarkable, blood pressure was normal, and no bruits were detectable over the carotid, subclavian, axillary, or renal arteries.

Routine laboratory examinations and immunological texts, including antinuclear, antineutrophil cytoplasmic, anticardiolipin, antibeta-2 glycoprotein 1 antibodies, and lupus anticoagulants were negative, with the exception of positive tuberculosis interferon-gamma release assay (QuantiFERON-TB Gold), suggesting latent *Mycobacterium tuberculosis* infection.

**Figure 1:**
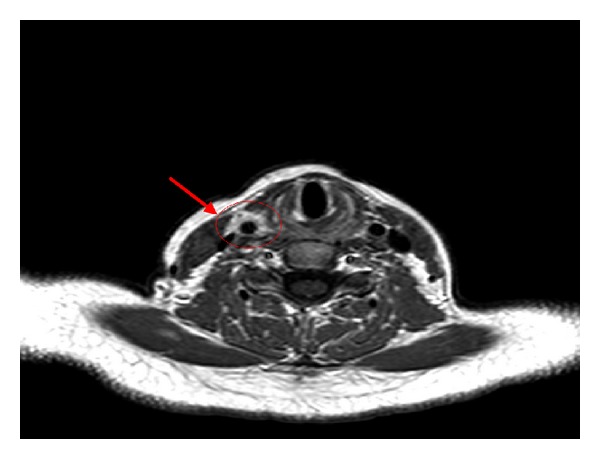
Magnetic resonance (MR) imaging of the neck. MR shows localized enhancement in T1-weighted images involving right medial-distal common carotid artery (arrow); the thickening of perivascular soft tissue might be the result of localized vascular inflammation.

The color-Doppler ultrasonography was performed to exclude possible arterial abnormalities; it showed an eccentric wall thickening of the distal common carotid artery without relevant hemodynamic effects and a suspected aneurysm. This latter was not confirmed by subsequent angiocomputed tomography, which also excluded cervical adenopathies, as well as abnormalities of adjacent tissues and supra-aortic vessels. To better evaluate the carotid alteration, we performed cranial, cervical, and thoracic magnetic resonance (MR), which showed only localized perivascular enhancement of the soft tissue in the right medial-distal common carotid artery in T1-weighted images, without intraluminal diameter variation (Figure 1). Furthermore, MR confirmed the absence of involvement of the aorta and its branches.

At this point the patient's clinical picture was classified as carotidynia and treated with nonsteroidal anti-inflammatory drugs, partially useful on pain that spontaneously resolved after three months.

## 3. Discussion

The clinical and imaging features observed in the patient here described were quite typical of carotidynia syndrome. In addition, careful clinicoserological and radiological investigations excluded well-known disorders potentially responsible for this syndrome; therefore, the present case can be classified as primary variant of carotidynia.

Of note, the vascular alterations revealed by MR were similar to those described in previous case reports [7–9], namely, a mural thickening of the carotid with abnormal gadolinium-enhancing tissue surrounding the distal tract of the vessel without intramural alterations.

In the absence of histopathological evaluation of the lesion, not performed because of its peculiar localization, the etiopathogenesis of carotid injury in the patient here described remains very difficult to explain. On the basis of the MR findings, it might be hypothesized that carotidynia is the expression of carotid alterations possibly due to localized vasculitic process or to very early stage of systemic arteritis (Takayasu's vasculitis?). In this respect, careful patient's clinical follow-up is necessary for a timely detection of potential disease progression and possibly to clarify the underlying pathogenetic mechanisms. At present, it is possible to hypothesize an immune-mediated process triggered by latent *Mycobacterium tuberculosis* infection, the same agent potentially involved in large vessel vasculitides, such as Takayasu's arteritis [13, 14]. The possible role of numerous infectious agents in the pathogenesis of vasculitides is largely investigated; these disorders are the results of multifactorial and multistep process. In this context, also latent *Mycobacterium tuberculosis* infection might represent a possible cofactor of large vessel arteritis; mycobacterial antigens may exert a chronic stimulus for the immune-system in genetically predisposed individuals, through different immunological modalities such as molecular mimicry mechanism [13, 14]. If further supported by the patient's clinical follow-up, the hypothesis of *Mycobacterium*-driven vasculitis might lead to interesting therapeutical implications such as the pre-emptive use of antitubercular treatment.
